# Short-term and long-term survival of patients with gastric cancer 

**Published:** 2021

**Authors:** Ali Karamoozian, Mohammad Reza Baneshi, Abbas Bahrampour

**Affiliations:** 1 *Department of Biostatistics and Epidemiology, Kerman University of Medical Sciences, Kerman, Islamic Republic of Iran*; 2 *Modeling in Health Research Center, Institute for Futures Studies in Health, Kerman University of Medical Sciences, Kerman, Islamic Republic of Iran*

**Keywords:** Gastric cancer, Short-term survival, Long-term survival, Cure rate frailty model, Bayesian inference.

## Abstract

**Aim:**

The aim of this study was to apply the Bayesian mixture cure rate frailty model to determine the factors that influence short-term and long-term survival of patients with gastric cancer

**Background:**

Determining the risk factors of gastric cancer is currently considered very important, because the disease has become one of the most dangerous types of mortal cancers. Therefore, it is possible to determine the effective risk factors of short-term and long-term survival in patients through utilizing this model.

**Methods:**

The present retrospective study was conducted on 339 gastric cancer patients whose data was recorded in hospitals of Kerman province, Iran, during 2001-2015. In the study, the Bayesian mixture cure rate frailty model was used to determine the effective factors of short-term and long-term survival in patients.

**Results:**

In the present study, the event of interest occurred for 57.5% of patients. Over time, the survival rate of cancer patients reached its lowest point, approximately 0.3 at the end of study. According to the results of the present study, variables of chemotherapy (β=-0.35 (-0.75, -0.03) and OR=1.59 (1.08, 2.19)), morphology (β =-0.98(-1.45, -0.48) and OR=2.99 (1.78, 4.17)), and metastasis (β =0.42(0.10, 0.93) and OR=0.39(0.01, 0.84)) were identified as effective factors in short-term and long-term survival of patients.

**Conclusion:**

The effective factors of long-term and short-term survival can be identified by utilizing the Bayesian mixture cure rate frailty model, while it is impossible through conventional models of survival analysis. Chemotherapy, morphology, and metastasis are the most important effective factors of short-term and long-term survival in patients with gastric cancer.

## Introduction

 Due to scientific advances in the treatment of some cancers, including gastric cancer, mortality as the event of interest does not occur for a considerable proportion of cancer patients or it occurs after a longer period of time. Such individuals are referred to as cured patients (1). From another perspective and considering the high lethality of some cancers in the late stages of the disease, it would be possible for a significant number of patients to be cured, or at least have a longer survival time, if the disease were to be diagnosed in its early stages (2). Therefore, cure rate models are a useful tool for analyzing cancer survival data.

In general, it is expected that all individuals will experience the event of interest when the follow-up is sufficiently long. However, this assumption sometimes is not the case. In the other words, a fraction of individuals do not experience the event of interest, even if they have been involved in the study for many years. Such individuals are referred to as cured patients in relation to the event of interest (3, 4). Conventional survival analysis models, such as the Cox regression model, cannot be used for data sets when a significant number of patients have been cured and the curing status is important. The assumption of such models is that all individuals have the same chance to experience the event of interest, and all of them will eventually experience it (5, 6). Therefore, the risk of an event is the same for cured individuals as it is for those who have not experienced the event of interest (7).

Cured individuals or individuals with long-term survival are considered in the analysis using cure rate models. The model also considers heterogeneity between individuals caused by their short-term and long-term survival rates. In this model, those individuals who experience the event of interest are referred to as the population with a short-term survival rate, and those who do not experience the event of interest are referred to as the population with a long-term survival rate (5). Among cure rate models, there are mixture cure rate models and non-mixture cure rate models (2,6). Generally, the type of mixture cure rate model is more commonly compared with the non-mixture cure rate model due to the simplicity of its theory. In this model, the results are presented for two groups of subjects in two different forms. The first group comprises cured individuals so that they will have odds of being cured. The other group includes those who have experienced the event of interest (2, 8). There is no principle on which one of these two models works best; it depends on their fitness levels with the data (2).

Sometimes, there is the possibility of heterogeneity between observations that can affect the results. This heterogeneity can results from either ignoring a number of important covariates or the correlation between observations (9). This heterogeneity causes varied frailty for different subjects. In other words, some individuals can experience the event of interest sooner than others. A significant difference between frailties of individuals can affect the results and lead to inaccurate estimations of coefficients and parameters (10, 11). To solve this problem, the frailty random variable is added to the mixture cure rate model to control this heterogeneity between observations.

The superiority of Bayesian inference over classical statistical inference lies in the fact that Bayesian inference can estimate more accurate results by defining a precise and specific distribution for parameters. Moreover, no significant change will occur in the obtained results by utilizing the Bayesian inference, even if the samples and values of the parameters are changed. Another superiority Bayesian inference has over classical statistical inference is that it can solve problems in relation to small sample sizes by adding the simulation of Markov chain Monte Carlo methods to this inference (12–15).

In recent years, the prevalence and death rates from gastric cancer have declined significantly in most parts of the world. However, this disease is the fourth most common cancer and is ranked second among mortal cancers after lung cancer. The survival rate of this cancer is higher in countries with a high prevalence compared to countries with a low prevalence (16, 17). However, it should be noted that the prevalence rates of this cancer vary among countries. It is possible to decrease the lethality of this cancer through appropriate therapeutic methods such as chemotherapy and a diet consisting of fruits and vegetables (17). On the other hand, studies have shown that mucosal incision has become one of the well-known treatments for gastric cancer in recent years. A literature review revealed that the 5-year survival rate of this cancer for all incidences equaled 20.7% before 1970, but significantly increased up to 28.4% before 1990. An in–depth review of incidences revealed that the survival rate increased from 37.6% to 55.4% in the same years (18). Therefore, utilizing new technologies and appropriate therapies are effective factors in improving the survival rate of gastric cancer.

Gastric cancer is one of the most common causes of mortality in developed and developing countries, including Iran where it is the third leading cause of death after heart disease and accident. In Iran, gastric cancer is the most common type of cancer among men, while it is the third most prevalent cancer among women. Since this cancer is mostly diagnosed in its latest stages, it has a relatively low survival rate. Studies conducted on some countries reported that the lowest and highest 5-year survival rates belonged to Thailand with 4.4% and the United States with 37%, respectively (9).

In general, the purpose of the present study was to determine the effective factors of short-term and long-term survival models among gastric cancer patients in Kerman province, Iran. For this purpose, the Bayesian mixture cure rate model was used taking into account the frailty random variable in this model. 

## Methods

This retrospective study was conducted from 2001 to 2015. The records of 846 gastric cancer patients who referred to Afzalipour Hospital and Shahid Bahonar Hospital of Kerman province in southeastern Iran were studied, and the information was then matched with the Kerman Cancer Registry Center database. Duplicate and unrelated cases of gastric cancer were removed, including information on other gastrointestinal cancers. The information of those patients who had an unknown death status was also discarded. The patients were followed up from 2001 to 2015, and ultimately, information on 339 gastric cancer patients became available after isolation and deletion of unrelated information. In the present study, the mixture cure rate model was used to analyze gastric cancer data due to the existence of data related to cured patients. As a number of effective factors of the model either were not measured or could not be identified, the frailty random variable was added to the model. Adding this variable to the model led to the more accurate estimation of results through controlling the heterogeneity between observations. On the other hand, the results of the model have been reported based on increasing or decreasing risk, considering the fact that the frailty model was applied on the frailty random variable based on conditional distribution of a proportional hazard structure. Unlike conventional models of survival analysis, the mixture cure rate model can determine the effective factors of patients with long-term survival in addition to determining the effective factors of their short-term survival. Therefore, the data was analyzed using a classical mixture cure rate model as well as the Bayesian mixture cure rate model while taking into account controlling the frailty effect by programming and executing the required instructions in R 3.5.1 statistical software.

**Covariates**

The dependent variable of the present study was time to event outcome. This variable includes the status of death due to gastric cancer or censoring the data of subjects (death or censor) as well as the time to death due to cancer or censoring the data of patients (month). The independent variables of the present study included age group (less or more than/equal to 60), gender (male/female), smoking history (no/yes), opium consumption (no/yes), place of residence (urban/rural), radiotherapy history (no/yes), chemotherapy history (no/yes), metastasis (no/yes), tumor morphology (neoplasm, carcinoma, or adenocarcinoma), histological grade (well, moderate, or poor) and cancer stage (I, II, III, or IV).

The variable of tumor morphology generally includes both carcinoma and adenocarcinoma. Carcinoma refers to the presence of malignant cells in the mucous membrane, also called cancer cells, that have not yet become cancerous tumors. If these malignant cells become cancerous, then adenocarcinoma can occur. Therefore, it can be said that adenocarcinoma is a more advanced and dangerous condition than carcinoma. However, the occurrence of either of these conditions in the stomach is often called gastric cancer. There is another condition for morphology called neoplasm, in which it is not exactly clear whether the mucosal cells are benign or malignant. In the present study, the three conditions of neoplasm, carcinoma, and adenocarcinoma were considered as levels of morphology.

The variables were classified according to their standard classifications and in consultation with a clinical expert.

**Statistical analyses**

First, the Kaplan–Meier survival curve was drawn for the existence of cured data. The survival curve became a smooth line and stable plateau at a point approximately 0.3 before reaching zero. After ensuring the existence of cured data, the mixture cure rate frailty model was used.

To evaluate the effect of variables on long-term survival, the survival function of the mixture cure rate model was used to calculate the odds ratio (OR) and its survival probability after determining the important and effective variables. 

The data was analyzed by introducing the appropriate distribution for time-to-event occurrence as well as the appropriate distribution for the frailty random variable in order to investigate the effect of variables on the short-term survival of patients. Weibull and gamma distributions are more applicable for time-to-event occurrence and the frailty random variable, respectively, compared to other distributions; therefore, these two distributions were used for modeling in the present study.

Posterior distribution must be determined in order to estimate the parameters and regression coefficients using Bayesian inference. The posterior distribution can be calculated through Bayes theorem and utilizing the likelihood function and prior distribution.

Finally, the regression coefficients were estimated by applying equations to the likelihood function and determining the posterior distribution using the Metropolis Hasting algorithm. Finally, the estimated values of parameters, standard deviation, and credible interval of 95% were reported for each of them.

**Ethical consideration**

Ethical approval was granted by the joint Ethical Committees of Kerman University of Medical Sciences and Modeling in Health Research Center (ethic no. IR.KMU.REC.1398.607).

## Results

The required data of the present study was related to 339 gastric cancer patients and gathered from hospitals of Kerman province, Iran. Among them, 42.5% of patients had censored status. Mean and standard deviation of observed times were 21.7 and 20.38 months, respectively. The median survival time was 25.46 months. The 3-year and 5-year survival rates were 0.41 and 0.32, respectively. Other information on covariates is presented in Table 1. 

**Figure1:**
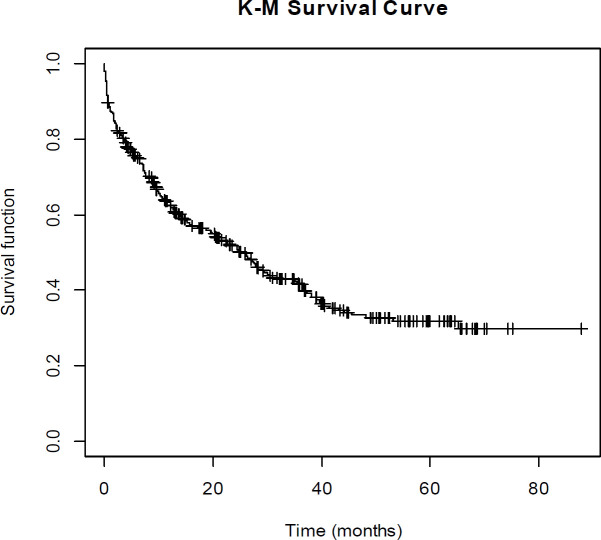
Kaplan-Meier curve for survival of patients with gastric cancer

The Kaplan–Meier survival curve was drawn to investigate the presence or absence of cured data in dataset. As seen in Figure 1, the Kaplan–Meier survival curve became a smooth line, and the stable plateau was observed at a point approximately 0.3 before reaching zero, indicating the presence of cured data in the dataset. Therefore, conditions were met for applying the mixture cure rate model.

**Table1:** Characteristics of risk factors for death-censored gastric cancer

Frequency (%)			Variables
Died	Censored		
121 (62.1)	95 (66)	Male	Sex
74 (37.9)	49 (34)	Female	
120 (61.5)	95 (66)	No	Opium
75 (38.5)	49 (34)	Yes	
142 (72.8)	106 (73.6)	No	Smoker
53 (27.2)	38 (26.4)	Yes	
161 (82.6)	111 (77.1)	Urban	Place of residence
34 (17.4)	33 (22.9)	Rural	
39 (20)	26 (18.1)	No	Surgery
156 (80)	118 (81.9)	Yes	
167 (85.6)	126 (87.5)	No	Radiotherapy
28 (14.4)	18 (12.5)	Yes	
117 (60)	87 (60.4)	No	Chemotherapy
78 (40)	57 (39.6)	Yes	
110 (56.4)	95 (66)	No	Metastasis
85 (43.6)	49 (34)	Yes	
176 (90.3)	134 (93.1)	No	Family History
19 (9.7)	10 (6.9)	Yes	
6 (3.1)	5 (3.5)	Well	Grade
151 (77.4)	119 (82.6)	Moderate	
38 (19.5)	20 (13.9)	Poor	
6 (3.1)	3 (2.1)	I	Stage
111 (56.9)	94 (65.3)	II	
56 (28.7)	35 (24.3)	III	
22 (11.3)	12 (8.3)	IV	
20 (10.3)	3 (2.1)	Neoplasm	Morphology
32 (16.4)	21(14.6)	Carcinoma	
143 (73.3)	120 (83.3)	Adenocarcinoma	
80 (41)	54 (37.5)	<60 year	Age Group
115 (59)	90 (62.5)	>=60 year	

The parameters and regression coefficients were estimated by determining the appropriate distribution as prior distribution of each parameter in Bayesian inference.

**Table2:** Estimates of parameter in models for short-term survivor

	Model with two-state morphology						Model with three-state morphology					
	Classical Model			Bayesian Model			Classical Model			Bayesian Model		
Parameters	Mean	SD	%95 Confidence interval	Mean	SD	%95 Credible interval	Mean	SD	%95 Confidence interval	Mean	SD	%95 Credible interval
β	2.40	1.86	(1.06, 7.89)	2.15	0.93	(1.09, 4.55)	2.47	1.89	(1.06, 8.22)	2.20	0.96	(1.09, 4.67)
γ_1_	1.01	0.02	(1.00, 1.07)	1.01	0.02	(1.00, 1.08)	1.01	0.02	(1.00, 1.07)	1.01	0.02	(1.00, 1.08)
γ_2_	2.70	1.96	(1.07, 8.09)	2.16	0.98	(1.09, 4.75)	2.67	2.06	(1.06, 8.02)	2.20	0.99	(1.08, 4.74)
Intercept	0.23	1.56	(-3.42, 4.96)	0.15	0.81	(-1.43, 1.74)	0.23	1.94	(-3.42, 4.96)	0.19	0.80	(-1.53, 1.88)
Chemotherapy												
Yes	-0.38	0.18	(-0.75, -0.04)	-0.35	0.17	(-0.70, -0.02)	-0.38	0.18	(-0.73, -0.05)	-0.35	0.17	(-0.70, -0.03)
No	0			0			0			0		
Morphology												
Neoplasm	-1.16	0.30	(-1.59, -0.68)	-0.98	0.28	(-1.44, -0.50)	-1.08	0.32	(-1.62, -0.53)	-0.98	0.23	(-1.45, -0.48)
Adenocarcinoma	0			0			0			0		
Metastasis												
Yes	0.54	0.23	(0.07, 1.11)	0.40	0.24	(0.09, 0.83)	0.52	0.25	(0.06, 1.13)	0.42	0.27	(0.10, 0.93)
No	0			0			0			0		

**Table3:** Estimates of odds ratio and survival probability for long-term survivor

	Model with two-state morphology				Model with three-state morphology			
	Classical Model		Bayesian Model		Classical Model		Bayesian Model	
Variables	OR (%95 CI)	Survival Probability (%95 CI)	OR (%95 CI)	Survival Probability (%95 CI)	OR (%95 CI)	Survival Probability (%95 CI)	OR (%95 CI)	Survival Probability (%95 CI)
Chemotherapy								
Yes	1.60 (1.04, 2.30)	0.68 (0.46, 0.83)	1.53 (1.04, 2.34)	0.66 (0.45, 0.82)	1.61 (1.08, 2.30)	0.69 (0.49, 0.83)	1.59 (1.08, 2.19)	0.66 (0.45, 0.81)
No	1	0.57 (0.45, 0.68)	1	0.56 (0.44, 0.66)	1	0.58 (0.47, 0.68)	1	0.55 (0.43, 0.66)
Morphology								
Neoplasm	3.43 (1.26, 4.57)	0.92 (0.71, 0.96)	2.99 (1.89, 4.17)	0.89 (0.74,0.95)	3.02 (1.86, 5.26)	0.91 (0.76, 0.97)	2.99 (1.78, 4.17)	0.89 (0.72, 0.95)
Adenocarcinoma	1	0.77 (0.66, 0.84)	1	0.73 (0.60, 0.82)	1	0.77 (0.63, 0.86)	1	0.73 (0.59, 0.82)
Metastasis								
Yes	0.25 (0.006, 0.88)	0.07 (0.0004, 0.38)	0.41 (0.02, 0.84)	0.14 (0.004, 0.36)	0.26 (0.003, 0.92)	0.08 (0.0002, 0.41)	0.39 (0.01, 0.84)	0.12 (0.004, 0.35)
No	1	0.23 (0.06, 0.41)	1	0.28 (0.13, 0.40)	1	0.25 (0.06, 0.43)	1	0.26 (0.10, 0.39)

**Table4:** Criteria for comparison between classical and Bayesian mixture cure rate frailty model

Criterion	Classical Model	Bayesian Model
DIC	1779.56	1698.56
EAIC	1815.70	1780.21
EBIC	1791.36	1755.86

DIC: Deviance Information Criterion; EAIC: Expected Akaike Information Criterion; EBIC: Expected Bayesian Information Criterion

As previously mentioned, data analysis was carried out separately for individuals with short-term and long-term survival through the mixture cure rate model. The results obtained from short-term and long-term survival of patients are presented in Tables 2 and 3, respectively. In Tableβ, $\gamma_{1}$ and $\gamma_{2}$ are parameters of the gamma and Weibull distributions that have been considered as frailty and baseline distribution parameters, respectively.

As can be seen from Table 2, the metastasis variable was significant in addition to the chemotherapy and morphology variables in both classical and Bayesian models. Patients with metastasis had a higher risk of mortality caused by gastric cancer compared to patients without metastasis. Patients who did not receive chemotherapy also had a higher risk of mortality caused by gastric cancer compared to those who received chemotherapy. In the term of morphology variable, it can be also argued that patients with adenocarcinoma status had a higher risk of mortality compared to patients with neoplasm status.

As can be seen in Table 3, the variables of chemotherapy, morphology, and metastasis were the effective factors of patients with long-term survival. Patients who received chemotherapy had higher odds of being cured compared to those who did not receive chemotherapy. In other words, those who received chemotherapy had 1.59-times better odds of being cured compared to those who did not receive chemotherapy, while the value of this ratio was equal to 1.61 in the classical model. In terms of the morphology variable, patients with neoplasm status had higher odds of being cured compared to patients with adenocarcinoma status. In other words, patients with neoplasm status had 2.99-times better odds of being cured compared to patients with adenocarcinoma status, while the value of this ratio was equal to 3.02 in the classical model. In terms of metastasis, it can be argued that this variable affected the cure rate of patients such that patients without metastasis status had 2.56-times better odds of being cured compared to patients with metastasis status, while the value of this ratio was equal to 3.85 in the classic model.

After model-fitting, it was found that there was a significant difference between neoplasm and adenocarcinoma statuses. According to the results, it can be claimed that the neoplasm cells were mainly benign, which brought about this significant difference. Therefore, a model with the same conditions as before, but with a two-state morphology variable instead of a three-state one, was fitted to confirm and interpret the results. In this model, the two levels of carcinoma and adenocarcinoma as malignant and neoplasm as benign were considered in two different groups. The differences in the results of both models were negligible; therefore, the results based on morphology with two types of grouping, two and three statuses, are presented in the appendix.

Table 4 represents the indicators used to comprise the fitness of classical and Bayesian models. As can be seen from the three indicators of fitness, the Bayesian model fits the data better than the classical model, because the values of these indicators were lower for the Bayesian model compared to the classical model. Considering the better fitness of the Bayesian model on data compared to the classical model, the results of the Bayesian model have been discussed.

## Discussion

About the model proposed in the present study, it can be argued that the cure rate model should be used when there is curing data in the dataset. In the present study, the Bayesian mixture cure rate model was used taking into account the frailty effect which is an appropriate and accurate model with respect to its current and applied distributions. It should be noted that the cured data is ignored in some studies, despite its presence, and common survival analysis models such as the Cox model are used in such studies. This is not correct, because the short-term survivors are ignored when the cured data is not considered, and it is assumed that all individuals will eventually experience the event of interest over time. However, there are individuals in the study for whom this assumption is not true. Therefore, the results obtained from these models are not accurate or precise (19). The cure rate model should be used in such studies in order to prevent this problem. On the other hand, it is recommended to control the frailty effect when utilizing the Bayesian inference in order to achieve accurate estimations of the coefficients and desirable results. The coefficients in the classical model were overestimated compared to the Bayesian model. Considering the better fitness of the Bayesian model compared to classical model, it is recommended to utilize the Bayesian model instead of the classical model to prevent the overestimation of coefficients.

In the present study, the variables of chemotherapy, morphology, and metastasis had a significant effect on long-term survival. Clearly, chemotherapy is one of the most common treatments for cancer. Given the high importance of this treatment, it is expected that patients who receive this treatment are either cured or survive longer (20–23). The cure rate can be increased in this model with this happening. The present study also found that chemotherapy increased the odds of a patient being cured. The variable of morphology is related to the size and shape of the cancerous tissue, which includes the three levels of neoplasm, carcinoma, and adenocarcinoma. The most primitive and least risky state is neoplasm, and the most dangerous one is adenocarcinoma (24). It was expected that patients with neoplasm morphology would have higher odds of being cured compared to patients with adenocarcinoma morphology. The reason for this is that some cells are benign and others are malignant in the neoplasm status, while all cells are malignant in the adenocarcinoma status (25). Therefore, the existence of benign cells can give patients higher odds of being cured. It can also be argued that metastasis occurs when cancer cells invade other organs of the body (26). When this happens, the odds of being cured is decreased due to the deteriorating condition of cancer cell control on one hand and the increased mortality risk caused by cancer on the other (27). Therefore, these variables can be considered as important for gastric cancer, and they should be taken into account in measures provided for patients with gastric cancer in order to increase their odds of being cured and decrease their mortality risk. In cure rate models, the curing odds not only refer to the low risk status of the desired variable, but also whether the variable has produced the cured data or not. In other words, variables are significant in long-term survival when their presence has provided the conditions for utilizing cure rate models where using conventional methods increase the probability of error in estimation.

The main issue in survival analysis is to examine the relationships between independent variables and patient survival rate and to determine what factors increase or decrease the risk of the desired event occurrence. Weibull distribution was used as the baseline distribution in the parametric model of the present study. The frailty random variable was also added to the model in order to take into account the hypothesis of proportional hazard and to provide more accurate results. In frailty models, modeling is applied on the frailty random variable based on conditional distribution of a proportional hazard structure (10). As with long-term survival, chemotherapy, morphology, and metastasis were the effective factors of short-term survival of patients. When a patient has metastasis, his/her condition is critical. Therefore, it can be expected that patients with metastasis are more likely to experience death caused by gastric cancer compared to patients without metastasis. The results of the present study also showed that the risk of death was higher for subjects with metastasis compared to subjects without it. It is expected that patients who receive chemotherapy will be less likely to experience the event of interest because of its high importance and curing of patients with this treatment. The status of adenocarcinoma for gastric cancer is also worse because of the presence of cancer cells in inner layers, including glands, compared to the neoplasm status, where some cells are not malignant (28). Therefore, patients with adenocarcinoma experience a higher rate of the event of interest. In the present study, other variables affected neither the short-term nor the long-term survival of patients.

The model used in this study is one of the strengths of this study as it considers the cure fraction. Moreover, using the Bayesian inference was a strength as it estimates more accurate results compared to the classical inference. Estimating the results for both short-term and long-term survival is another strength of this study. The incompleteness of the medical records of some patients may be the weakness of this study, but with the use of Bayesian inference, the effect of this weakness was minimized.

In general, it can be concluded from the results of the present study that Bayesian mixture cure rate frailty models should be used in the presence of cured data. The superiority of Bayesian inference over classical inference is in the fact that Bayesian inference can resolve the concern about constant values of distribution parameters in the model. In other words, results will be more desirable by changing the data and carrying out new sampling which may have different parameters. The reason is that Bayesian inference assumes an appropriate distribution for each parameter with a wide range of values instead of constant parameter estimation. Furthermore, the overestimation of coefficients can be prevented by utilizing the Bayesian model instead of the classical one. 

The variables of chemotherapy, morphology, and metastasis are also important factors that have a significant effect on the short-term and long-term survival of patients. Finally, the findings of the present study showed that chemotherapy is an appropriate and important treatment for cancer patients. According to the morphological results, it is recommended to pay serious attention to the growth, proliferation, and propagation of cells from early stages of the disease in order to decrease the risk of patient death as much as possible. This matter can also prevent the occurrence of metastasis as one of the high risk factors for gastric cancer death.
